# Thrombotic thrombocytopaenic purpura in the era of HIV: A single-centre experience

**DOI:** 10.4102/sajhivmed.v24i1.1504

**Published:** 2023-10-27

**Authors:** Yusuf Moola, Zaheera Cassimjee, Chandni Dayal, Sheetal Chiba, Adekunle Ajayi, Malcolm Davies

**Affiliations:** 1 Department of Internal Medicine, Faculty of Health Sciences, University of the Witwatersrand, Johannesburg, South Africa; 2 Division of Nephrology, Helen Joseph Hospital, Johannesburg, South Africa

**Keywords:** TTP, HIV, plasma exchange, mortality, renal dysfunction

## Abstract

**Background:**

Thrombotic thrombocytopaenia purpura (TTP) is a rare disorder which carries a high mortality. HIV is an important cause of TTP.

**Objectives:**

We assessed the presentation and response to plasma exchange (PEX) by HIV status.

**Method:**

A single-centre retrospective review of all patients receiving PEX for TTP between 01 January 2010 and 31 December 2019 was undertaken. Demographics and presenting parameters were compared between HIV-associated TTP and other aetiologies using Mann-Whitney *U* and Kruskal Wallis analysis of variance testing, as appropriate. The effect of aetiology and presenting parameters on PEX duration was modelled using Cox proportional hazards; effect of these variables on mortality and residual renal dysfunction in survivors was analysed using stepwise multivariate regression.

**Results:**

Uncontrolled HIV infection was the commonest cause (81.9%) of TTP in the 83 patients identified. Thrombocytopaenia was more severe and neurological deficit more frequent in HIV-associated TTP; but renal dysfunction was milder in this group. Aetiology did not influence mortality risk. Aetiological category and presenting parameters did not predict PEX duration. Residual renal dysfunction was less frequent in survivors of HIV-associated TTP.

**Conclusion:**

HIV is an important cause of TTP in the local context. Haematological and neurological involvement are more severe in HIV-associated TTP. Acceptable survival rates are achievable with PEX even in advanced HIV infection; renal sequalae are less common in this group.

**What this study adds:** Analysis of this large cohort expands knowledge of the clinical features of HIV-associated thrombotic thrombocytopaenia purpura. Despite an aggressive presentation and association with uncontrolled HIV infection, mortality when treated with plasma exchange does not appear to be significantly higher than other forms, and residual renal dysfunction in survivors may be rare.

## Introduction

The thrombotic migroangiopthies (TMA) are a group of disorders of heterogenous aetiology with a shared pathology featuring endothelial injury and microvascular occlusion by platelet-rich thrombi.^1^ Consumptive thrombocytopaenia and microangiopathic haemolytic anaemia are ubiquitous and defining features of TMA.^1,2^ Downstream target organ hypoperfusion caused by microvascular occlusion manifests in a spectrum which includes varying degrees of neurological and renal dysfunction, clinically distinguished into thrombotic thrombocytopaenic purpura (TTP) or haemolytic uraemic syndrome (HUS).^2^

Thrombotic migroangiopthies is a rare disorder in the developed world, with an estimated annual incidence of four cases per million population.^3^ South African case series implicate HIV as an important cause of TTP in the local context, with people living with HIV (PLWH) comprising 77% – 78.8% of patients diagnosed with the disorder.^4,5,6^ Analysis of plasma exchange (PEX) prescribing patterns by the South African National Blood Service (SANBS) suggests an annual incidence of HIV-associated TTP of 17.6–63.8 cases per million population.^7^ Although less common in international series, HIV is nevertheless disproportionately represented as an aetiological factor in TTP in countries with low population prevalence rates of HIV. For example, the French Network on TMA has reported that HIV accounted for 12.3% of cases in their setting.^8^

Although the pathophysiology of HIV-associated TTP has yet to be fully elucidated, several mechanisms have been proposed. These include endothelial dysfunction from chronic inflammation or endothelial injury caused by shed HIV viral proteins and complement activation,^9^ direct infection of endothelial cells by the virus,^10^ and the development of autoantibodies to ADAMTS13 (a disintegrin and metalloproteinase with a thrombospondin type 1 motif, type 13), an enzyme which exerts an antithrombotic effect through the cleavage of ultra-large von Willebrand factor (UL-vWF).^11^

Previous case series suggest a higher mortality rate in cases of HIV-associated TTP, possibly reflecting an increased risk of infectious complications arising from the immunoparesis associated with advanced HIV infection, which is a characteristic association of the disorder.^12^ Delays in diagnosis, caused by the wide differential diagnosis for TMA in PLWH, may further compromise the outcome of HIV-associated TTP.^2^

Residual renal dysfunction is an under-reported outcome parameter in most TMA series. Available data suggest considerable variation in the prevalence of chronic kidney disease (CKD) between aetiological categories of TMA.^13^ Overall, up to 45% of TMA survivors may manifest new-onset hypertension, and approximately 21% demonstrate some degree of loss of glomerular filtration rate (GFR), with between 3% and 18% requiring ongoing kidney replacement therapy (KRT).^14^ While TTP has been reported to be a significant cause of acute kidney injury (AKI) in PLWH in the South African context,^15^ evidence for the contribution of HIV-associated TTP to kidney disease in the local context is lacking.

We report the largest series of cases of HIV-associated TTP from South Africa to date. Analysis of these data provides a better characterisation of the presentation of the disorder which may facilitate improved diagnosis and earlier initiation of treatment among clinicians treating PLWH – a vital step in improving outcomes. We further show that treatment of HIV-associated TTP with PEX is associated with reasonable survival outcomes, and that residual renal dysfunction in this disorder is fortuitously rare.

## Methods

A retrospective single-centre review of all suspected cases of TTP treated with PEX during the period 01 January 2010 – 31 December 2019 was undertaken. The overall prevalence rate of HIV in the local community served by this centre has been estimated to be 12.9%;^16^ institutional surveillance has reported HIV prevalence rates of 42.4% in medical admissions.^17^ Retrospective confirmation of the diagnosis of TTP was made in consideration of accepted guidelines and previous case series definitions; in brief, all included patients manifested evidence of consumptive thrombocytopaenia (platelet count below 140 × 10^9^/mm^3^) and microangiopathic haemolytic anaemia (schistocytes on peripheral blood film with serum lactate dehydrogenase activity above 190 IU/mL) and had an international normalised ratio (INR) below 1.5.^2,6,18^ Standard therapy for TTP at this centre is PEX with emergent antiretroviral (ARV) initiation in cases of HIV-associated TTP; immunosuppression is reserved for cases of autoimmune disease-related TTP as recommended by guidelines.^2^

Data were anonymously extracted from clinical case records and stored in an Excel (Microsoft Corp., Redmond, Washington, United States) spreadsheet which was imported into Statistica version 14.0.1 (TIBCO Software, Palo Alto, California, United States) for analysis; Stata version 17 (StataCorp, College Station, Texas, United States) was used to plot remission curves. Estimated GFR (eGFR) was recalculated from presenting serum creatinine using the CKD-EPI formula with the race coefficient removed. Median haemoglobin concentration, platelet count, lactate dehydrogenase (LDH) activity level, creatinine concentration, eGFR at presentation, and the presence of neurological deficit or pyrexia were described for the series as a whole and for HIV infection categories; the Mann-Whitney U and Fisher exact tests were used to compare parameters between categories. The effect of presenting parameters and HIV infection status on the duration of PEX required to induce remission was modelled using Cox regression analysis censored for inpatient death. In-hospital mortality and residual renal dysfunction (defined as an eGFR of less than 60 mL/min per 1.73 m^2^ on discharge) outcomes were modelled using stepwise multivariate logistic regression analysis.

### Ethical considerations

This study was undertaken in adherence with the Declaration of Helsinki. Permission to undertake the study was obtained from the Human Research Ethics Committee of the University of the Witwatersrand (protocol number M2011116).

## Results

A total of 166 cases were reviewed, of which 64 were excluded for retrospectively missing data; a further 11 patients were excluded due to retrospective review indicating the possibility of a diagnosis other than TTP. A further three patients were excluded due to the possibility of pregnancy-related haemolysis elevated liver enzymes, and low platelets (HELLP) syndrome, with an additional three patients excluded due to the presence of overlapping TTP aetiological categories (two HIV-positive patients with HIV-associated malignancies, and one patient known with systemic lupus erythematosus who presented with newly diagnosed HIV infection); a further two patients were excluded on the basis of age less than 18 years at presentation. The final cohort included in analysis therefore comprised 83 patients, including 68 PLWH and 15 HIV-negative patients.

Presenting features of the cohort are described in Table 1. Women of black African ethnicity constituted the largest patient group in this series (52 patients, 62.7% of the cohort). Renal dysfunction was not uncommon, with a presenting eGFR below 60 mL/min per 1.73 m^2^ documented in 30 cases (36.1% of the cohort); documented altered neurological function and pyrexia were rarer manifestations of TTP in retrospective analysis (26.5% and 7.2% of the cohort). HIV was the dominant aetiological factor for TTP in this series, accounting for 81.9% of cases.

**TABLE 1:** Presenting features of thrombocytopaenia purpura.

Characteristic	*n*	%	Median	Interquartile range
Age (years)	-	-	36	32–43
**Sex**				
Female	56	67.85	-	-
Male	27	32.5	-	-
**Ethnicity**				
Black African people	78	94.0	-	-
Non-black people	5	6.0	-	-
Mixed ethnicity people	2	2.4	-	-
Asian people	2	2.4	-	-
White people	1	1.2	-	-
Haemoglobin (g/dL)	-	-	6.9	5.9–8.4
Platelet count (×10^9^/dL)	-	-	15	12–22
LDH (IU/mL)	-	-	1460	866–1958
Creatinine (mmol/L) ?	-	-	98	68–132
CKD-EPI eGFR (mL/min per 1.73 m^2^)	-	-	83	47–105
Neurological deficit	22	26.5	-	-
Pyrexia	6	7.2	-	-
**Aetiology**				
HIV	68	81.9	-	-
Autoimmune disorder	9	10.8	-	-
Systemic lupus erythematosus	7	8.4	-	-
Mixed connective tissue disease	2	2.4	-	-
Idiopathic	6	7.2	-	-
**HIV-positive patients**				
CD4 count (×10^6^/mm^3^)	-	-	46.5	80.5–211
Viral load (copies/mL)	-	-	212 000	72 864–425 980
Viral load < 20 copies/mL	0	0	-	-
Antecedent ART prescription	12	17.6	-	-

CKD-EPI, chronic kidney disease epidemiology collaboration; eGFR, estimated glomerular filtration rate; ART, antiretroviral therapy; LDH, lactate dehydrogenase.

Presentation with HIV-associated TTP was associated with uncontrolled HIV infection. Only 12 patients (17.6%) had been initiated onto antiretroviral therapy (ART) prior to presentation, and no patient included in this series had a suppressed viral load at presentation with TTP. CD4 count was low in this series.

Platelet count was lower, but renal function was better preserved in PLWH presenting with TTP compared to HIV-negative patients; neurological deficit was more frequently encountered in PLWH (Table 2) and PLWH were less likely to require dialysis for TTP-related AKI than HIV-negative patients (9.1% of PLWH required dialysis compared to 53.8% of HIV-negative patients; odds ratio for dialysis for PLWH 0.09, 95% confidence interval [95% CI]: 0.02–0.34, *P* < 0.001).

**TABLE 2:** Presenting features of thrombocytopaenia purpura compared between HIV infection groups.

Characteristic	HIV-positive patients (*n* = 68)				HIV-negative patients (*n* = 15)				*p*
	*n*	%	Median	Interquartile range	*n*	%	Median	Interquartile range	
Age (years)	-	-	35.5	32–43.5	-	-	36	27–42	0.277*
Haemoglobin (g/dL)	-	-	6.8	5.7–8.3	-	-	7.4	7.2–8.6	0.148*
Platelet count (×10^9^/dL)	-	-	14	11–19	-	-	24	14–50	0.013*
LDH (IU/mL)	-	-	1483	975.5–1950.5	-	-	1279	449–1984	0.327*
eGFR (mL/min per 1.73 m^2^)	-	-	84	53.5–105.5	-	-	33	10–101	0.025*
Neurological deficit	21	30.9	-	-	1	4.6	-	-	0.046**
Pyrexia	4	5.9	-	-	2	13.3	-	-	0.296**

eGFR, estimated glomerular filtration rate; LDH, lactate dehydrogenase.

* , determined by Mann-Whitney U test;

** , determined by Fisher Exact test.

**FIGURE 1:**
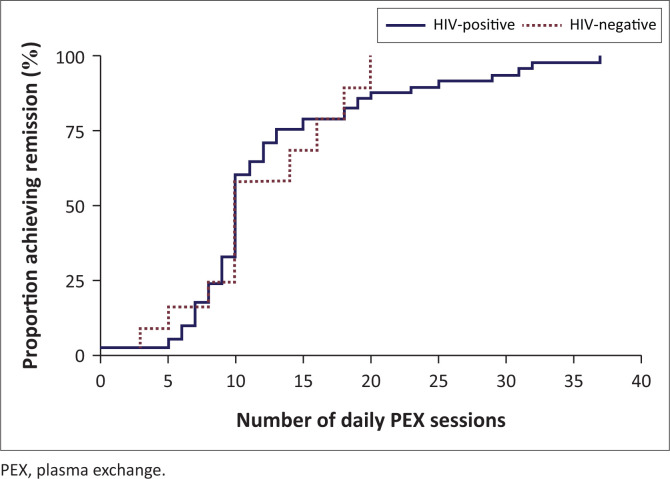
Time to remission in survivors compared between HIV infection groups.

The crude inpatient mortality rate was not significantly different between HIV-positive and HIV-negative patients in this series (Table 3). There was no difference in the number of PEX sessions required to induce remission of TTP (Figure 1), nor was there a significant difference in the total duration of hospitalisation in survivors between these patient groups. Median eGFR on discharge was not significantly different between HIV-positive and HIV-negative survivors of TTP; however, eGFR below 60 mL/min per 1.73 m^2^ on discharge was more frequent in the latter group.

**TABLE 3:** Outcome parameters compared between HIV infection groups.

Characteristic	HIV-positive patients				HIV-negative patients				*p*
	*n*	%	Median	Interquartile range	*n*	%	Median	Interquartile range	
Crude inpatient mortality rate	-	10.3	-	-	-	26.7	-	-	0.106*
Number of PEX sessions to induce remission	-	-	10	9–13	-	-	10	-	0.963**
Duration of hospitalisation in survivors (days)	-	-	15	13–25	-	-	21.5	-	0.416**
eGFR (mL/min per 1.73 m^2^) on discharge	-	-	102	85–115	-	-	72	-	0.147**
Percentage of survivors with eGFR below 60 mL/min per 1.73 m^2^ on discharge	6	9.8	-	-	4	36.4	-	-	0.039*

PEX, plasma exchange; eGFR, estimated glomerular filtration rate.

* , determined by Fisher Exact test;

** , determined by Mann-Whitney U test.

No effect was detected for presenting haemoglobin concentration (*P* = 0.402), platelet count (*P* = 0.572), LDH activity (*P* = 0.866), eGFR (*P* = 0.584), or HIV infection status on the number of PEX sessions required to induce remission; among PLWH no effect was observed for presenting CD4 count (*P* = 0.920) or HIV viral load (*P* = 0.800) on this parameter.

A significant effect for renal function at presentation was observed on the risk of inpatient mortality and on the risk of reduced renal function as defined by eGFR below 60 mL/min per 1.73 m^2^ on discharge (Table 4). No effect was observed for presenting haemoglobin concentration, platelet count, LDH activity, HIV infection status, or (among PLWH) presenting CD4 count or viral load on these two outcomes.

**TABLE 4:** Effect of presenting parameters on mortality and renal outcomes.

Outcome	Odds ratio	95% confidence interval odds ratio	*p*
**Inpatient mortality**			
Haemoglobin (g/dL)	1.29	0.87–1.93	0.201
Platelet count (×10^9^/dL)	1.00	0.96–1.04	0.867
LDH (IU/mL)	1.00	0.99–1.00	0.489
eGFR (mL/min per 1.73 m^2^)	0.96	0.94–0.99	0.003
HIV-positive	0.99	0.16–6.03	0.987
CD4 count (×10^6^/mm^3^)	0.92	0.56–1.49	0.723
Viral load (copies/mL)	1.00	0.99–1.00	0.563
**Residual renal dysfunction (eGFR < 60 mL/min per 1.73 m^2^ on discharge)**			
Haemoglobin (g/dL)	1.23	0.81–1.89	0.334
Platelet count (×10^9^/dL)	0.97	0.92–1.02	0.232
LDH (IU/mL)	1.00	0.99–1.00	0.318
eGFR (mL/min per 1.73 m^]^)	0.94	0.91–0.97	< 0.001
HIV-positive	0.21	0.03–1.44	0.113
CD4 count (×10^6^/mm^3^)	0.99	0.98–1.01	0.727
Viral load (copies/mL)	1.00	0.99–1.00	0.620

eGFR, estimated glomerular filtration rate; LDH, lactate dehydrogenase.

## Discussion

This large cohort confirms reports from previous series of the importance of uncontrolled HIV infection as an aetiological factor for TTP in the local context (Table 5). HIV-associated TTP presents with haematological manifestations of greater severity and is more frequently associated with neurological deficit than other forms of the disorder, although renal function appears better preserved in the former. Response of HIV-associated TTP to PEX and emergent ARV initiation in the present study is reassuring, with the duration of PEX required to induce remission being comparable to other aetiologies. In addition, HIV-associated TTP in this study had a superior survival rate and lower rate of residual renal deficit.

**TABLE 5:** Previous studies of HIV-associated thrombotic microangiopathies.

Reference	Study design	*n*	Clinical presentation	Outcomes reported
**South African data**				
Novitzky et al.^19^ (Cape Town)	Single-centre cohort	21 (23 HIV-controls)	HIV-TTP dominant in black women Lower haemoglobin, platelet count in HIV-TTP Neurological deficit more frequent in HIV-TTP No difference in creatinine Low CD4 count in HIV-TTP	HIV-TTP highly responsive to plasma infusion Lower mortality rate in HIV-TTP (5% vs 21%)
Gunther et al.^20^ (Johannesburg)	Single-centre cohort	22 (3 HIV-controls)	Lower haemoglobin, platelet count in HIV-TTP	Not reported
Louw et al.^4^ (Johannesburg)	Single-centre cohort	16 (4 HIV-controls)	Significant female preponderance in HIV-TTP TTP was the presenting feature of HIV infection in 47%	96.5% survival rate with PEX Platelet count, LDH, and degree of immunosuppression do not predict duration of PEX required for remission
Swart et al.^5^ (Johannesburg)	Single-centre cohort	32 (14 HIV-controls)	HIV-TTP dominant in black women HIV-TTP associated with advanced infection	Overall mortality rate 29.3% High rates of refractory TTP in PLWH (76.9%) Advanced HIV disease predicts refractory TTP Neurological deficit and advanced HIV predict mortality
Masoet et al.^6^ (Stellenbosch)	Single-centre cohort	40 (12 HIV-controls)	HIV-TTP dominant in black women Pyrexia more frequent in HIV-TTP No difference in haemoglobin, platelet count, renal function, or neurological deficit Low CD4 count and low rates of antecedent ART prescription in HIV-TTP	HIV-TTP highly responsive to plasma infusion Similar remission rates to HIV-negative Similar mortality rate to HIV-negative (44.2% vs 43.9%)
**International data**				
Gervasoni et al.^21^	Single-centre cohort (Italy)	17	HIV-TTP dominant in men HIV-TTP associated with advanced infection	100% mortality in HIV-TTP prior to ART
Becker et al.^22^	Multicentre cohort (US)	17	HIV-TTP dominant in men HIV-TTP associated with advanced infection	Increased risk of mortality in HIV-TTP
Miller et al.^23^	Single-centre cohort (UK)	8	HIV-TTP dominant in black women	100% survival with PEX and ART initiation
Malak et al.^8^	Multicentre cohort (France)	29 (62 HIV-controls)	No difference in haemoglobin, platelet count, or neurological deficit Better preserved renal function in PLWH	Higher mortality in HIV-TTP associated with higher ADAMTS13 activity levels
Hart et al.^24^	Multicentre cohort (UK)	24	HIV-TTP dominant in black women HIV-TTP associated with advanced infection	Low mortality rate (4%) High viral load predicts longer duration of PEX required to induce remission
Bade et al.^25^	Single-centre cohort (US)	28 (74 HIV-controls)	HIV-TTP more common in black patients HIV-TTP associated with advanced infection Platelets more frequently lower in HIV-TTP No difference in renal dysfunction	No difference in mortality rate

Note: Please see the full reference list of the article for more information.

TTP, thrombocytopaenia purpura; PEX, plasma exchange; PLWH, people living with HIV; ART, antiretroviral therapy; PLWH, people living with HV; US, United States; UK, United Kingdom; ADAMTS13, disintegrin and metalloproteinase with a thrombospondin type 1 motif, member 13.

The prevalence of HIV-associated TTP as an aetiological category within the TMAs is known to be high in South Africa, accounting for approximately 78% of cases in the previous series.^4,5,6^ Lower rates of HIV-associated TMA in international series may, in part, reflect greater access to ART in the developed world; reflecting this, a decrease in the incidence of the disorder has been reported in these series since the advent of widespread ART access.^21^

Women and patients of black African descent are over-represented in thrombotic microangiopathy registries. Low frequency of HLA-DRB1*04, a human leukocyte antigen subtype known to confer protection against TMA, has been implicated as contributing to an inherited risk for the disorder in people of black African ethnicity.^26^ Mechanisms underlying the female preponderance of patients diagnosed with TMA may include gender discrepancies in autoimmunity^27^ and the effect of oestrogenic hormones on endothelial function and vWF production.^28^ Disparities arising from gender and ethnic inequalities in South Africa place the greatest burden of HIV infection on young women of black African ethnicity.^29^ The tendency of HIV-associated TTP to afflict young black women is evidenced by this and other South African series.^5,6,19^

Variations in the clinical presentation of TMA between aetiological categories may reflect differences in comorbidities associated with the underlying primary disease process. For example, lower haemoglobin concentration and platelet count in HIV-associated TTP observed in this and other series^19, 20,25^ may reflect comorbid HIV-related dyshaematopoiesis.^9^ More frequent neurological deficit in HIV-associated TTP observed in this and other series^19^ may be due to TTP-mediated exacerbation of cognitive disorders prevalent in PLWH, or may reflect a predilection by TMA for central nervous system involvement as a feature of the neurotropic tendencies of the virus.^30^ Poorer renal function in HIV-negative patients in this series may reflect the substantial number of cases of systemic lupus erythematosus (SLE) in this group (7 cases, 46.7% of the HIV-negative cohort), with the known association of TMA with proliferative glomerulonephritis^31^ accounting for more severe renal involvement in these patients.

The use of PEX as a first-line therapy in this series follows recommended treatment guidelines^2^ and is predicated upon theoretical benefits over plasma infusion (PI), including removal of autoantibody and facilitation of a greater volume of plasma and hence ADAMTS13 replacement. Although previous studies suggest PEX superiority in response rates,^32^ local series have reported favourable outcomes with PI alone.^6,19^ The time to remission using PEX in the present study (10 days) is similar to that reported by Masoet et al. in their series using PI (11 days)^6^ and to that reported by Louw et al. in their series using PEX (12 days).^4^ In the absence of severe renal dysfunction which may limit the volume of plasma that can safely be infused, the similarity in duration of therapy suggests that PI alone may be a reasonable treatment strategy in settings where PEX is unavailable.^6,19^ As noted by Louw et al., presenting features of TMA in this series are poor predictors of the duration of therapy required to induce remission.^4^

Previous case series suggest a higher mortality rate in cases of HIV-associated TTP, possibly reflecting an increased risk of infectious complications arising from the immunoparesis associated with advanced HIV infection.^12^ More recent South African series have reported no difference in survival in PLWH,^6,19^ which may reflect the universal prescription of ART as a component of TMA treatment. Initiation of ART in the present series may account for the lack of effect observed for presenting CD4 count and HIV viral load on survival outcomes.

Renal dysfunction has been reported to predict mortality in TMA in other studies.^33^ Prescription of dialytic support as indicated for the management of TTP-related AKI reduces the probability of a direct effect for renal dysfunction in inpatient mortality in the present series. Instead, the severity of renal dysfunction may evidence risk of other organ involvement, in particular the risk of acute myocardial infarction, which is a known significant contributor to TTP inpatient mortality.^34^

Renal dysfunction is an under-reported outcome parameter in most TTP case series. Available data suggest considerable variation in the prevalence of residual renal dysfunction between aetiological categories.^13^ Analysis of inpatient outcomes in the present series suggests that significant residual renal dysfunction in survivors of HIV-associated TTP is uncommon. As partially evidenced in limited duration follow-up in this cohort, the severity of AKI correlates with the probability of residual renal dysfunction and, thus, with CKD.^35^ Concerns over the contribution of HIV-associated TTP to AKI in the local context^15^ may be unfounded, and the disorder may carry a lower risk for CKD than that reported for other TTP aetiologies.^14^ Further follow-up is, however, required to better characterise long-term renal outcomes in survivors of TTP in this and other series.

There are limitations to the present study. Although guidelines recommend ADAMTS13 measurement as part of the evaluation of the aetiology of TMA,^2^ resource constraints do not permit routine measurement at our institution. Levels of ADAMTS13 activity and of associated inhibiting autoantibodies in the setting of HIV-associated TMA appear to show considerable variability,^11^ and reduced ADAMTS13 activity and autoantibodies have been detected in PLWH in the absence of TMA.^36^ For these reasons, the PLASMIC (platelet count, combined haemoLysis variable, Active cancer, absence of stem-cell or solid organ transplant, mean corpuscular volume [MCV], international normalised ratio [INR], creatinine) score, which predicts the probability of severe ADAMTS13 deficiency and hence may assist in confirming the diagnosis of TTP, is not routinely applied in HIV-associated TTP. We also acknowledge the lack of kidney histological evaluation in patients with severe renal dysfunction, against which background an adequate explanation for the association of autoimmune-mediated TMA with poorer eGFR at presentation through concomitant proliferative glomerulonephritis must remain hypothetical. This lack of biopsy material reflects clinical concerns as to the risk of haemorrhage in patients with thrombocytopaenia and uraemia. An additional limitation is the lack of long-term follow-up of renal outcomes in this cohort. We further acknowledge that the retrospective nature of this study is likely to have compromised the accurate recording of observational data such as neurological deficits. Finally, the single-centre nature of this study could limit the generalisability of our findings. We would, however, suggest that the application of a consistent diagnostic and treatment algorithm, enabled by the single-centre nature of this study, facilitates a more accurate analysis of presentation and outcomes.

## Conclusion

Our study demonstrates the significant contribution of HIV to the incidence of TTP in the South African context. HIV-associated TTP in the local setting demonstrates a predilection for young women of black African ethnicity, likely reflective of genetic and sex-related risk and of the demographics of HIV infection in South Africa. Variation in the clinical presentation of TTP may reflect comorbidities associated with underlying aetiological factors. Despite more severe haematological parameters and neurological deficit in HIV-associated TTP at presentation, response to PEX is similar to cases of TTP occurring in HIV-negative patients. Survival rates with PEX are comparable to those reported internationally, even in the setting of advanced HIV infection. Residual renal dysfunction in survivors of HIV-associated TTP appears rare, but further long-term studies are required to better evaluate this under-reported complication.
